# Early effects of COVID-19 on physical activity and screen time use among elementary school students in Columbus, New Mexico

**DOI:** 10.1016/j.dialog.2022.100053

**Published:** 2022-09-29

**Authors:** Juan C. Padilla, Jill A. McDonald, Christopher Sroka, Cynthia Kratzke, Jagdish Khubchandani

**Affiliations:** a Department of Public Health Sciences, College of Health, Education & Social Transformation, New Mexico State University, PO Box 30001, Las Cruces, NM 88003-8001, USA; b Southwest Institute for Health Disparities Research, College of Health, Education & Social Transformation, New Mexico State University, PO Box 30001, Las Cruces, NM 88003-8001, USA; c Department of Economics, Applied Statistics and International Business, College of Business, New Mexico State University, PO Box 30001, Las Cruces, NM 88003-8001, USA

**Keywords:** Physical Activity, Screen Time Use, Child Health, Hispanic Health, Rural Health, COVID-19

## Abstract

Adherence to national physical activity guidelines among youth ages 6-11 in the United States is low. The emergence of COVID-19 and the public health measures implemented in response may have decreased children’s physical activity even further. We conducted an online survey among parents of students attending Columbus Elementary School in Columbus, New Mexico, a rural community on the US-Mexico border, to assess changes in children’s physical activity and screen time use from summer 2019 to summer 2020. We also sought to identify important covariates. All parents (N = 55) and children (N = 87) identified as Hispanic; most parents were born in Mexico, while most children were born in the United States. Most parents (79.3%) reported a decrease in their children’s physical activity from 2019 to 2020, and the vast majority of these parents reported that the changes were due to COVID-19 home confinement. The mean number of days children were physically active for >60 minutes significantly decreased, while daily screen time use increased. Having parents born in Mexico, infrequent family meals (<3/week), and not having community spaces for physical activity close by protected children from decreases in their level of physical activity from 2019 to 2020. Home-based exercise may serve as a suitable method of physical activity when public health responses to COVID-19 restrict community spaces. Future interventions should also be mindful of the role that parental nativity and related cultural factors may play in children’s physical activity levels.

## Highlights

• Physical activity decreased and screen time use increased from 2019 to 2020
• Parents perceived COVID-19 as the reason why children’s activity levels decreased
• Home factors such as infrequent family meals led to smaller decreases in PA
• Lack of access to community spaces was associated with a smaller decrease in PA

## 1 Introduction

Regular physical activity is one of the most important behaviors for promoting good health, improving cardiovascular fitness, preventing chronic conditions like obesity, and supporting the development of long-term healthy exercise habits among youth [1]. In support of these health benefits, the Physical Activity Guidelines for Americans *2*^*nd*^ *edition* recommend that youth 6–17 years old engage in ≥60 minutes of moderate-to-vigorous physical activity daily [1]. However, adherence to this guideline in the United States is low, and even lower among Hispanic youth (21.9%) when compared to non-Hispanic White youth (24.9%) [2].

Children’s physical activity is influenced by several factors. Children in rural communities and communities of low socioeconomic status are typically less active than others [3]. Positive parent and child perceptions of the neighborhood environment are associated with increased physical activity [4]. Existing research also suggests that parental social support, parenting strategies such as verbal encouragement, and parental activity patterns may increase Hispanic children’s physical activity [5]. In Texas *colonias*, low-income US-Mexico border communities lacking adequate infrastructure, neighborhood safety concerns such as unleashed dogs, heat, traffic, and absence of streetlights have been reported to limit children’s physical activity [6,7]. More physical activity barriers in these *colonias* have been reported during summer compared to the school year [8]. Children’s physical activity may also be threatened by the COVID-19 pandemic; home confinement due to the pandemic has been reported to negatively affect physical activity globally [9].

As of September 23, 2022, over 616,600 cases and 8,500 deaths due to COVID-19 have been reported in New Mexico [10]. While important in reducing the transmission of COVID-19, initial public health measures implemented by New Mexico - limiting access to in-person learning facilities, recreational areas, and youth sports, as well as lifestyle adjustments to the pandemic - may have decreased youth physical activity [11,12]. Studies in other areas of the world have produced strong evidence to support this assessment. For example, children in Spain - one of the first countries to impose a nationwide COVID-19 lockdown in Europe - had significant decreases in physical activity levels and increases in screen time use during the lockdown [[13], [14], [15], [16], [17]]. However, to date, no studies have assessed the impact of COVID-19 on children’s physical activity and screen time use in New Mexican border *colonias*. Initial disruptions to physical activity, if any, may have long-lasting consequences to children’s health in these communities. As the incidence of new COVID-19 cases fluctuates with the emergence of new variants, disruptions to everyday life in New Mexico continue; some schools, for example, reverted to online learning during the Omicron wave [18]. The possibility of future disruptions continue to put children’s ability to engage in adequate physical activity at risk, which is of special concern in New Mexico’s vulnerable *colonias*.

### 1.1 Purpose

Guided by a socioecological framework, we sought to assess children’s physical activity during the initial stages of the pandemic in Columbus, New Mexico through multiple levels of influence and assess COVID-19’s role in any observed changes. Columbus, a Spanish-speaking *colonia* with a population of 1,442, is located approximately four miles from the Mexico border [19]. The purpose of our study was to measure the physical activity of elementary school-aged children in Columbus in summer 2019 and summer 2020. In addition, we aimed to measure children’s screen time use during those periods and to identify factors associated with changes in physical activity and screen time use. Assessing COVID-19’s impact on children’s physical activity in Columbus and identifying factors that could modify activity levels will help inform future interventions and build community resilience to future disruptions in physical activity due to new COVID-19 variants and other infectious diseases.

## 2 Methods

In collaboration with Columbus’ only elementary school, Columbus Elementary School, we conducted a cross-sectional study among parents and/or guardians of students enrolled in grade levels K-5 in December 2020. One hundred twenty-one students enrolled in the school resided in Columbus during the study period (Viridiana Sanchez [school principal], personal communication, November 8, 2020). School staff had active phone-to-phone communication with all the parents of these students. Some kindergarten children were below the age of 6, but all children in the study were assessed using the physical activity guidelines for youth 6-17 years old. All study procedures were reviewed for human subjects concerns and approved by the authors’ university institutional review board.

### 2.1 Recruitment

Permission to conduct an online survey of parents in Columbus Elementary School was provided by the school administration and the Deming Public Schools District. School administrative staff coordinated study recruitment efforts which included 24 teachers and 25 classrooms. An initial invitation and up to two weekly reminders to participate in the study were sent out to each student’s parent/guardian by their classroom teacher. Teachers sent the survey link via text message to those who consented to receive the survey. Families with multiple children attending the school received multiple invitations but were instructed by teachers to complete the survey only once because the survey covered all children in their household in grades K-5. The online survey was created using Qualtrics© and was piloted by two school administrative staff members before its finalization. Parents had the option of completing the survey in English or Spanish on either a personal computer or smartphone. Study materials, including the study consent form and survey, were consistent with an 8^th^-grade reading level.

### 2.2 Study variables

The online survey included questions about demographic and behavioral characteristics and household norms. We included variables that had been used in previous studies of Hispanic and US-Mexico border region youth [[4], [5], [6], [7], [8],^20^], some of which were observed to be associated with our outcome measures. Demographic characteristics included each child’s age, sex, ethnicity, country of origin (nativity), and primary language. We included figures of child silhouettes to measure parental perception of each child’s weight and classify children by their weight status [21]. We also collected parental demographic characteristics including education level, height, and weight.

Household characteristics included income, size, and 10 household norms [[4], [5], [6], [7],^20^]. We asked parents whether they took away outdoor playtime or electronics as punishments for child misbehavior, whether electronics were the only way to keep their children entertained, the frequency of being active together as a family, the difficulty of finding time to play outside with their children, whether it was safe for their children to be physically active outdoors, and whether their children were more likely to be physically active if they were physically active. We asked questions about mealtime norms, including whether the TV was on during meals, the use of electronics during meals, and the frequency of family meals. We also asked parents if there were community spaces close by (“reasonably accessible” in terms of distance) for their children to be physically active.

The Godin Leisure-Time Exercise Questionnaire (GLTEQ) and the physical activity question from the National Survey on Children’s Health (NSCH) were used to assess students’ average weekly physical activity in summer 2019 and summer 2020. The GLTEQ is a three-item instrument designed to measure sessions of vigorous, moderate, and light physical activity lasting for at least 15 minutes [22]. The physical activity question from the NSCH, “During THE PAST WEEK, on how many days did this child exercise, play a sport, or participate in physical activity for at least 60 minutes,” was used to measure adherence to the physical activity guideline that children be active for ≥60 minutes daily [23]. These questions, and one on daily screen time use [20], were included for two time points: summer 2019 and summer 2020. Questions to determine the parental perception of change in their children’s physical activity from summer 2019 to summer 2020 and the perceived reason for any change were also included in the survey. In total, the survey had 36 questions.

### 2.3 Analysis

Descriptive statistics were calculated for all question responses. A total weekly leisure activity score was calculated from the three GLTEQ items. Under the GLTEQ, a ≥15-minute session of strenuous exercise was scored ‘9’, moderate exercise ‘5’, and mild/light exercise ‘3’ [22]. The sum of all exercise session scores is the weekly leisure activity score: a score of 24 or more was classified as *active*, 14-23 as *moderately active*, and <14 as *insufficiently active*. Heavy screen time use was defined as ≥4 hours/day [20].

Adjusted mixed-effects regression models were used to determine changes in the mean GLTEQ score, the number of active days/week (days/week with > 60 minutes of physical activity), and the number of hours/day of screen time from summer 2019 to summer 2020 and to identify covariates associated with changes in these outcomes. Children were clustered by their family unit using these models. Twenty covariates consisting of child and parent demographics, household characteristics, and household norms were first tested individually with our outcome measures. All covariates with *p* values <0.25 were included in the final models. Response categories with <10 observations were collapsed when possible. Four variables were excluded from the models due to <10 observations in a category: child nativity, parental primary language, whether parents took away electronics as punishment for child misbehavior, and whether children were more likely to be physically active if the parent was active. A “missing” category was created for three variables that had more than 10 missing observations: parental BMI, parental education, and household income. Study analyses were conducted using IBM SPSS Statistics Grad Pack 27.0.

## 3 Theory

**Fig. 1:**
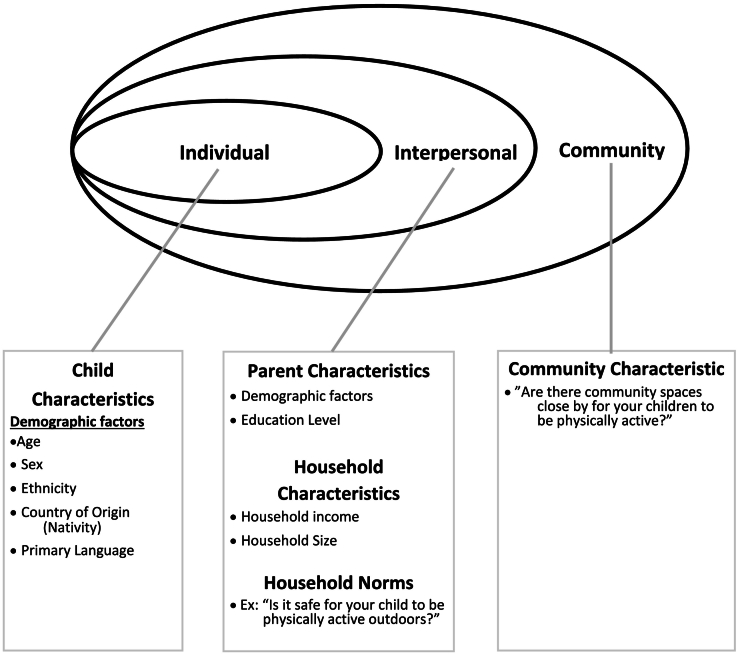
Framework for the study of changes in physical activity among children, Columbus, NM, 2019–2020.

According to the socioecologic framework, health outcomes are shaped by factors at various levels of influence [24]. Our research considers three levels of influence for children’s physical activity: individual (child characteristics); interpersonal (parental and household influences); and community (Fig. 1). The community level of influence is addressed by determining children’s access to community spaces for physical activity. Classifying factors according to these three levels is important in order to identify target areas for physical activity interventions.

## 4 Results

Fifty-three mothers, one father, and one grandmother completed the survey. Eighty-seven children (72%) of the 121 children in school were included in our final analyses. Four additional parents accessed the survey but did not complete it. Thirty-two (58.2%) of the surveys were completed in Spanish.

### 4.1 Child and parent demographics

All respondents identified themselves and their children as Hispanic (data not shown). More students were boys (55.4%) than girls, the vast majority were born in the US (96.3%), and most had parents who were born in Mexico (60%) (Table 1). Over 80% of children and parents spoke Spanish as their primary language. The mean age for children was 7.7 years and for parents 32.7 years. Parents reported most children as having a “normal” weight (80.6%). Among the parents who reported their height and weight, 63.4% were classified as “overweight” (BMI 25-29.9) or “obese” (BMI > 30). Three of every four households in the study had an income of $30,000 or less.

**Table 1:** Characteristics of the study population - Columbus, NM.

Characteristic	Category	*n*	%
Child characteristics			
Child Gender	Male	46	55.4
	Female	37	44.6
Child Age	4–7 years	38	46.9
	8–11 years	43	53.1
Child Ethnicity	Hispanic	81	100
Child Nativity	United States	79	96.3
	Mexico	3	3.7
Child Primary Language	English	13	16.2
	Spanish	67	83.8
Child Weight Status	Underweight	9	12.5
	Normal weight	58	80.6
	Overweight	5	6.9
	Obese	0	0
Parental/household characteristics			
Survey Language	English	23	41.8
	Spanish	32	58.2
Respondent relationship to Children	Mother	53	96.4
	Other	2	3.6
Parental Age	22–30 years	23	41.8
	31–53 years	32	58.2
Parental Ethnicity	Hispanic	55	100
Parental Nativity	United States	22	40.0
	Mexico	33	60.0
Parental Primary Language	English	6	10.9
	Spanish	49	89.1
Parental BMI	Underweight (<18.5)	3	7.3
	Normal weight (18.5–24.9)	13	31.7
	Overweight (25–29.9)	13	31.7
	Obese (≥30)	12	29.3
Parental Education Level	Less than high school	14	26.4
	High school graduate	22	41.5
	At least some college/trade profession	17	32.1
Household Income	<$10,000	17	34.0
	$10,000–$20,000	10	20.0
	$20,000–$30,000	11	22.0
	>$30,000	12	24.0
Household Size	2–4	27	51.9
	5–7	25	48.1
Household Norms			
When your child misbehaves, do you ever take away his/her outdoor play time? (n=54)	No	46	85.2
When your child misbehaves, do you ever take away his/her electronics? (n=54)	Yes	51	94.4
Does it ever seem the only way to keep your child entertained is to encourage his/her use of TV, tablet, video games, or other electronics? (n=53)	No	28	52.8
How many times a week does your family do active things together? (n=55)	Never	4	7.3
	1 or less	15	27.3
	2–3	28	50.9
	>3	8	14.5
Is it hard for you to find time to play outside with your child? (n=55)	No	31	56.4
Is it safe for your child to be physically active outdoors? (n=54)	Yes	37	68.5
If you’re physically active, is it more likely for your child to also be active? (n=53)	Yes	49	92.5
Is the TV on when your child eats? (n=54)	Yes	27	50.0
When eating together as a family, is there anyone who uses electronics? (n=55)	No	39	70.9
During a normal week, how often does your family eat a meal together? (n=55)	Never	0	0.0
	1 or less	0	0.0
	2–3	10	18.2
	>3	45	81.8
Community characteristic			
Are there places close by for your child to be physically active? (n=54)	Yes	37	68.5

Note: Only child weight status, child age, and parental BMI had more than 10 missing responses; the remaining child characteristics did not have more than 10 missing responses; the remaining parental characteristics and household norms did not have more than 5 missing responses

### 4.2 Household norms and community spaces

Almost half of the parents (47.2%) reported that encouraging the use of electronics is the only way to keep their children entertained and 92.7% reported doing at least one active thing together per week as a family. A majority indicated that it was not hard to find time to play outside with their children (56.4%) and that it was safe for their children to be physically active outdoors (68.5%). All parents reported that their families ate ≥2 meals/week together, with 81.8% eating ≥3 meals/week together. More than two-thirds of parents (68.5%) indicated that there were community spaces close by for their children to be physically active.

### 4.3 Change in physical activity from Summer 2019 to Summer 2020

Nearly four of every five parents indicated their children’s physical activity decreased from 2019 to 2020 and the vast majority of those indicated the change was due to COVID-19 home confinement (Table 2). On average, children’s GLTEQ score decreased by 12.0 points (95% confidence interval [CI], −27.5 – 3.5) and children spent almost one less day/week (−0.9) being active for ≥60 minutes (95% CI, −1.6 – −0.2). On average, children spent 1.5 more hours/day using electronics (95% CI, 0.8 – 2.2) in 2020 than in 2019.

**Table 2:** Physical activity and screen time use in the study population (summer 2019 to summer 2020) – Columbus, NM

Physical Activity Measures	Category	*n*	%			
Change in physical activity from summer 2019 to summer 2020	Decreased	65	79.3			
	Increased	5	6.1			
	No change	12	14.6			
Reason for change in physical activity	COVID-19 home confinement	72	87.8			
	Making lifestyle changes	3	3.7			
	Did not change	6	7.3			
	Not specified	1	1.2			

				2019 Mean	2020 Mean	Change Mean (95% CI)

GLTEQ Score^a^				67.5	46.3	−12.0 (−27.5 – 3.5)
Active Days/Week (≥60 minutes)^b^				4.0	2.9	−0.9 (−1.6 – −0.2)
Screen Time Use (Hours/day)^c^				3.2	5.0	1.5 (0.8 – 2.2)

Measure is based on the Godin Scale Classification [22]^a^; Measure is based on the physical activity question used in the National Survey on Children’s Health [23]^b^; Measure is based on the definition of heavy screen time [20]^c^

### 4.4 Associations with a change in physical activity

Children whose parent was born in the US had a larger decrease in their GLTEQ score from summer 2019 to summer 2020 (−36.0; 95% CI, −6.4 – −5.7) than children whose parent was born in Mexico (Table 3). Children in families who ate <3 meals/week together had a smaller decrease in their GLTEQ score (47.0, 95% CI, 4.0 – 90.0) than children in families that ate ≥3 meals/week together. Children who had community spaces for physical activity close by had a larger decrease in the number of active days/week (-1.9; 95% CI, −3.5 – −0.2) compared to children without nearby recreational spaces. No associations were found for changes in daily screen time use.

**Table 3:** Mixed-effects regression models for changes in physical activity and screen time use in the study population, adjusted for demographics, household characteristics, and household norms (summer 2019 to summer 2020) – Columbus NM

Covariates	Category	Outcome Variable		
		GLTEQ Score (95% CI)	Active Days/Week (≥60 minutes) (95% CI)	Daily Screen Time Use (≥4 hours) (95% CI)
Parental Nativity	Mexico	*Reference*		
	United States	**−36.0 (−66.4– −5.7)**		
Parental BMI	Missing	*Reference*		*Reference*
	Underweight and normal weight	−1.8 (−41.4 – 37.8)		1.7 (−0.4 – 3.8)
	Overweight	−5.1 (−50.3 – 40.0)		0.5 (−1.9 – 2.9)
	Obese	4.7 (−39.4 – 48.9)		0.8 (−1.4 – 3.0)
Household income	Missing			*Reference*
	<$10,000			0.8 (−2.3 – 4.0)
	$10,000–$20,000			−0.1 (−3.3 – 3.1)
	$20,000–$30,000			1.4 (−2.1 – 4.8)
	>$30,000			0.2 (−3.2 – 3.7)
Age, Parent		1.8 (−1.0 – 4.5)		0.0 (−0.1 – 0.2)
Age, Child			−0.02 (−0.1 – 0.0)	
Is your child entertained primarily through electronics?	No	*Reference*	*Reference*	
	Yes	−31.4 (−72.5 – 9.7)	−0.9 (−2.7 – 0.9)	
How often does your family do active things together?	>3 times		*Reference*	
	≤1 time		−0.9 (−3.6 – 1.7)	
	2–3 times		−1.3 (−3.4 – 0.8)	
Is it hard for you to find time to play outside with your child?	No	*Reference*		*Reference*
	Yes	−21.6 (−56.7 – 13.5)		0.9 (−0.7 – 2.4)
How many times does your family eat together during the week?	≥3 times	*Reference*		*Reference*
	<3 times	**47.0 (4.0– 90.0)**		0.6 (−1.5 – 2.6)
Is it safe for your child to be active outside?	No		*Reference*	
	Yes		1.2 (−0.6 – 2.9)	
Are there places close by for your child to be physically active?	No		*Reference*	
	Yes		**−1.9 (−3.5–−0.2)**	

Note: Results in bold font are statistically significant at *p* <.05; positive estimate indicates greater value than reference category; negative estimate indicates lesser value than reference category; only covariates with p <.25 in the individual tests were included in the models.

## 5 Discussion

Our study examined the effect that COVID-19 had on children’s physical activity and screen time use in a New Mexican border *colonia*. Results show that the number of active days/week among children attending elementary school in Columbus, New Mexico decreased significantly from summer 2019 to summer 2020. Over 95% of the parents who reported a drop in their children’s physical activity attributed the drop to COVID-19 home confinement resulting from public health restrictions. During this same period, parents reported that their children’s screen time use increased significantly. Children with a parent born in Mexico, without community spaces close by, and who experienced infrequent (<3) weekly family meals were found to be at lower risk of decreased physical activity in summer 2020 than other children; no associations were found for changes in screen time use.

Results show that children in our study had low levels of physical activity compared to children in New Mexico before the COVID-19 pandemic; only 20% met the physical activity guideline of being active ≥60 minutes daily in summer 2019 compared to 27% of all New Mexico children in 2016 [25]. In summer 2020, <10% of our cohort met this guideline. Since most parents who reported a decrease in their children’s physical activity believed the drop was due to COVID-19 home confinement, it is possible that the restrictions put in place in New Mexico to protect the public from COVID-19 limited opportunities to be physically active for children in Columbus, as observed elsewhere. Indeed, these results support a growing body of literature that suggest COVID-19 restrictions had a negative effect on the physical activity of children in the United States and communities around the world [11,[26], [27], [28], [29], [30]]. The pandemic may have also been perceived as a neighborhood safety threat by parents and children that discouraged the use of recreational spaces in the community.

Consistent with the socioecologic framework, protective factors were observed in association with changes in children’s physical activity at the interpersonal and community levels. At the interpersonal level of influence, having a parent/guardian born in Mexico was found to protect against decreases in children’s GLTEQ score. Research suggests Hispanic mothers with high acculturation levels have lower physical activity than mothers with low acculturation [31]. Nativity is often used as a proxy measure for acculturation, but its validity is disputed partly for conflating lived experiences and effects inherited from the previous generation [31]. Our findings here suggest that children’s physical activity may be influenced in part by their parent’s nativity, supporting the claim that the experiences of one generation may reflect in the health outcomes of the following generation [32].

Additionally, our findings suggest that infrequent family meals/week protected against decreases in children’s GLTEQ score. Although family meals and physical activity have both been shown to protect against obesity, previous research suggests that these demands compete with one another given the time pressures’ in parents’ daily lives [33]. It is possible that during the pandemic, some parents in our study chose to prioritize their children’s physical activity over family meals.

At the community level of influence, not having community spaces close by protected against decreases in children’s number of active days/week. It is possible that the closure of recreational areas as a COVID-19 safety measure had greater negative impacts on children who had these resources nearby and were accustomed to using them for physical activity. Children who did not live close to these resources possibly engaged in other forms of physical activity, such as home-based exercise, and were able to sustain these forms of activity during the pandemic. Indeed, families with outdoor spaces, such as backyards and driveways, have reported greater engagement in outdoor physical activity among their children compared to families without outdoor spaces in their homes; families with lower income were less likely to have outdoor spaces in their homes compared to families with higher income [34]. Consistent with this research, children in Brazil with large outdoor spaces at their homes had higher levels of physical activity compared to children with small or no outdoor spaces at their homes during the COVID-19 pandemic [35]. Home-based exercise may be an effective way of maintaining adequate physical activity during the pandemic when the use of outdoor recreational areas is limited [36].

Parents in this study reported that their children’s screen time use increased by 1.5 hours/day from summer 2019 to summer 2020 and the percentage of children who engaged in heavy screen time use (≥4 hours/day) more than doubled. In a previous study conducted in Columbus in 2016 [20], approximately 15% of children engaged in heavy screen time use on weekdays and >25% did so on weekend days; in our study heavy weekly screen time use was greater in 2019 and in 2020. It is possible that COVID-19 exacerbated screen time use trends among children in Columbus, which may already have been on an upward trajectory. While our study did not assess why children used electronic devices, prior work has shown that homework and recreational activities such as video games were the most common reasons for using electronics. The increased screen time use observed in our study could not have been due to educational purposes because the study time points were in the summer and summer school was not provided in 2020 (Viridiana Sanchez [school principal], personal communication, November 8, 2020). Regardless of the reason, increased screen time use has several health implications. Children in our cohort may be more likely to engage in heavy screen time use as adolescents and be more likely to have anxiety and sleeping disturbances as a result [17,37]. Increased screen time use has also been associated with increased weight status of children and possibly lower physical activity [38]. In our study, children’s active days decreased as their hourly screen time increased in 2020.

To our surprise, no associations were found between the covariates we studied and changes in screen time use; several associations had been previously documented between household norms and children’s screen time use in Columbus [20]. While not completely understood, certain child and parental demographic factors may be interacting with these norms. Indeed we found that parents with an “overweight” or “obese” BMI classification were more likely to let someone in their household use electronics during meals compared to parents without an “overweight” or “obese” BMI classification. Furthermore, children who primarily spoke English were more likely to use electronics during meals than children who primarily spoke Spanish. We also found that parents born in Mexico were more likely to have the television on during their children’s meals than parents born in the US, and parents with a high school education or less were more likely to believe that the use of electronics is the only way to keep their children entertained than parents with at least some college education. Thus, these associations warrant more research to better understand the relationships between household norms and demographic factors and their influence on children’s physical activity and screen time use.

Based on the figures of child silhouettes, <10% of children in our study were reported to be overweight or obese compared to >25% of Hispanic children in the US [39]. It is possible that parents underreported their children’s weight status in our study. Researchers who developed and tested the child silhouettes for Hispanic children ages 3–5 reported that mothers using these figures tended to perceive their children as thinner than their actual size [21]. Since the children in our study ranged from ages 4–11, these figures may not have provided accurate measures for older children. However, when considering only children ages 4–5, only 1/14 was reported to be overweight, suggesting that underreporting did play a factor in children’s BMI classification.

Several study limitations must be noted. First, our study may have excluded children in Columbus who dropped out of school due to COVID-19 and those who were either home-schooled or attended school outside of Columbus. However, school administrators indicated that the vast majority of elementary school-aged children in Columbus receive their education in the local elementary school. School administrators also reported a negligible drop-out rate due to COVID-19. Second, our study excluded Columbus Elementary School students who resided in Puerto Palomas, Mexico, Columbus’ sister community directly across the border, due to extensive communication challenges between faculty and parents during school closures. Additionally, although we had a strong response rate, missing responses and the small sample size created challenges when exploring associations between covariates and our outcome measures; it is possible that some potentially important covariates may have been excluded from the adjusted mixed-effects regression models. Finally, parental self-report measures and long recall periods may have introduced bias in our study data.

This study has multiple strengths. To the best of our knowledge, this study is the first to assess the parental perception of the effect that COVID-19 had on children’s physical activity in a US-Mexico border *colonia.* Secondly, we obtained a strong response rate (over 72% of eligible students), which can be attributed to the support of Columbus Elementary School faculty and staff and highlights the importance of community partners in community-based research. Lastly, the use of an online survey was effective despite Internet access concerns in rural areas [40]. Using an online survey and emphasizing completion through a smartphone, as this study did, may be an effective way to collect data from rural communities in future research. Data from the American Community Survey shows that residents in rural areas in the US had greater access to smartphones (79.5%) than a desktop or laptop (73.6%) in 2018 [41]. This finding supports previous research suggesting that rural border communities like Columbus may have greater Internet access than expected [20].

### 5.1 Conclusion

The COVID-19 pandemic has negatively affected human health in a multitude of ways, contributing to a staggering global disease burden, loss of life, and increasing social isolation. To the best of our knowledge, our study is the first to assess parent’s perception of the effect of COVID-19 on children’s physical activity levels in the US-Mexico border region. We provide evidence that children’s physical activity decreased and screen time use increased during the pandemic. Most parents believed this change in behavior to be a result of COVID-19 lockdown measures. More research is needed to determine the longevity of these changes in behavior; it is possible that these changes have persisted since lockdown measures were eased. Existing research on this topic is mixed; some studies suggest that children’s physical activity and screen time use did not revert to pre-pandemic levels after schools were reopened, while others suggest that physical activity measures returned to their baseline upon students returning to school [28,42]. Additional research is needed in Columbus to determine children’s physical activity levels post-lockdown and to better understand the implications of these changes on children’s long-term health. Hispanic children living in rural communities are already at higher risk for childhood obesity than other children; a lack of physical activity due to COVID-19 may exacerbate these risks and lead to a myriad of health issues such as sleeping problems, irritability, feelings of sadness and overall poorer health [17,39,43].

Results of this study point to the important influence of home and community environments on children’s physical activity. Future interventions that promote children’s physical activity should consider incorporating home-based exercise and be mindful of the time pressures faced by families in their daily lives. Cultural norms associated with parental nativity and family response to pandemic safety measures also warrant further study. Our results can inform efforts to reverse changes in children’s physical activity levels due to the COVID-19 pandemic and increase the resiliency of border communities against the negative effects of future public health threats.

## Funding

This research did not receive any specific grant from funding agencies in the public, commercial, or not-for-profit sectors.

## Availability of data and material

Study data will not be available due to the small sample size and concerns with the identification of participants.

## Declaration of Competing Interest

The authors declare that they have no known competing financial interests or personal relationships that could have appeared to influence the work reported in this paper.
